# Growth of few-wall carbon nanotubes with narrow diameter distribution over Fe-Mo-MgO catalyst by methane/acetylene catalytic decomposition

**DOI:** 10.1186/1556-276X-7-102

**Published:** 2012-02-02

**Authors:** Vladimir A Labunov, Alexander S Basaev, Boris G Shulitski, Yuriy P Shaman, Ivan Komissarov, Alena L Prudnikava, Beng Kang Tay, Maziar Shakerzadeh

**Affiliations:** 1 Belarusian State University of Informatics and Radioelectronics, P. Brovki 6, Minsk 220013, Republic of Belarus; 2 SMC (Technological Centre), Zelenograd, Moscow 124 498, Russia; 3 School of Electrical and Electronic Engineering, Nanyang Technological University, Singapore 639798, Singapore

## Abstract

Few-wall carbon nanotubes were synthesized by methane/acetylene decomposition over bimetallic Fe-Mo catalyst with MgO (1:8:40) support at the temperature of 900°C. No calcinations and reduction pretreatments were applied to the catalytic powder. The transmission electron microscopy investigation showed that the synthesized carbon nanotubes [CNTs] have high purity and narrow diameter distribution. Raman spectrum showed that the ratio of G to D band line intensities of *I*_G_/*I*_D _is approximately 10, and the peaks in the low frequency range were attributed to the radial breathing mode corresponding to the nanotubes of small diameters. Thermogravimetric analysis data indicated no amorphous carbon phases. Experiments conducted at higher gas pressures showed the increase of CNT yield up to 83%. Mössbauer spectroscopy, magnetization measurements, X-ray diffraction, high-resolution transmission electron microscopy, and electron diffraction were employed to evaluate the nature of catalyst particles.

## Introduction

Carbon nanotubes [CNTs] due to their incredible properties have attracted scientific and practical interest for almost 20 years [1]. High electrical and thermal conductance, striking mechanical strength, and an especially unique chirality-dependence electronic structure make CNTs one of the most promising alternatives to replace some of today's materials used in microelectronics manufacturing. In particular, due to an enormous field enhancement factor and high conductivity, CNTs are perfect sources for electron emission [2]. Tremendous numbers of attempts have been made to use carbon nanotube arrays as the field-emission cathodes [FECs] [3]. Single-wall carbon nanotubes [SWNT] provide a large field enhancement factor, low threshold voltage, and high emission currents, but the substantial degradation of emission currents is a serious bottleneck for the application of SWNT-based FECs [4]. In contrast, multi-wall carbon nanotubes [MWNTs] have high emission stability but have a small field enhancement factor. Few-wall carbon nanotubes [FWNTs] are considered as an ideal choice for this application [5]. Despite the progress in producing high-impurity and high-yield few-wall CNTs [6], the cost and simplicity of CNT fabrication still remain as the major problems limiting their wide application. In general, the main methods of CNT synthesis are arc discharge, pulsed laser ablation, and chemical vapor deposition [CVD]. CVD method has been recognized as the most suitable for commercial production of CNTs. The use of large specific surface powder with catalytic sites distributed on it is the common approach for high-yield CNT processes. Fabrication of a catalyst for synthesis of CNTs with desired geometrical properties (number of walls) is the art of today's carbon nanotube technology.

So far, bimetallic catalysts are accepted as the most efficient for high-yield production of CNTs. Metal alloys such as Co-Ni [7], Fe-Co [8,9], Fe-Mo [6,10], and Co-Mo [11,12] were utilized in catalysts for CNT growth. There are a number of works discussing the transition metals: molybdenum ratio and role of molybdenum in the efficient growth of CNTs with uniform diameter distribution [13-16]. A high amount of molybdenum in Fe-Mo alloy leads to the growth of larger-diameter CNTs, and vice versa, decreasing the Mo content in bimetallic alloy favors smaller-diameter CNT synthesis. This fact most likely relates to the catalytic particle size dependence on Fe_x_Mo_y_O_z _phases from which these particles are released during the reduction process; in turn, oxide phase formation depends on calcination (annealing) processes [17].

Usually, hydrogen serves as a reduction agent for catalyst activation. Variation of hydrogen flow during the reduction step and hydrogen/hydrocarbon ratio during the synthesis affects the quality and purity of the CNT product [18]. On the other hand, transition metal catalysts themselves are known as promoters of methane decomposition for high-rate hydrogen conversion [19]. It allows us to avoid the use of any additional reducing agent for catalyst activation.

Simplification of the high-yield production method and reducing the cost of catalyst components are two crucial steps towards the commercialization of the FWNT product. In this paper, we describe the developed technological approach for a simple and efficient FWNT synthesis.

### Experiment

**Figure 1:**
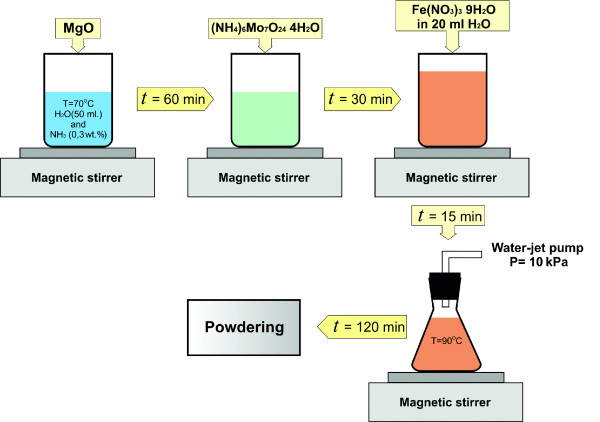
**The scheme of the catalyst preparation**.

Fe-Mo-MgO catalyst (molar ratio 1:8:40) was prepared by impregnation method. The scheme of catalyst preparation is presented in Figure 1. Ferric nitrate (Fe(NO_3_)_3_·9H_2_O), ammonium heptamolybdate ((NH_4_)_6_Mo_7_O_24_·4H_2_O), and MgO of 'purum' quality were chosen as starting materials. Since the purification step is very critical for potential applications of CNTs, MgO was used as a support because the reaction products of MgO with mineral acids can be easily dissolved in water. MgO powder (2 g) was steeped in a 50-ml water solution of NH_3 _(0.3 wt.%), and the suspension was subsequently stirred at 70°C for 60 min. Then, 0.88 g of ammonium heptamolybdate was added to the suspension, and it was stirred for 30 min. Lastly, 20 ml of a water-based solution of ferric nitrate Fe(NO_3_)_3_·9H_2_O (1.02 g) was infused, and the suspension was stirred additionally for 15 min. Adding of Fe(NO_3_)_3_·9H_2_O leads to the formation of Fe(OH)_3 _clusters, and as a consequence, the color of the suspension turns orange. The powder mixture was dried by a water-jet pump under the pressure of 10 kPa at 90°C for 120 min. After drying, the catalyst powder was ground in an agate mortar. No further calcination was applied.

Finally, the catalyst was placed in a quartz boat and then directly loaded into the hot zone of a 15-mm tubular furnace at the temperature of 900°C while the furnace was purged with 100 cm^3^/min of Ar. When the temperature was stable, 40 cm^3^/min flow of methane was introduced. After 15 min, the CH_4 _flow was set to zero simultaneously introducing 10 cm^3^/min of acetylene flow. The CNT growth process was stopped by switching off the C_2_H_2 _flow, and the product was immediately removed from the hot zone and cooled down to the room temperature at 300 cm^3^/min of Ar flow which took about 5 min. The synthesis of CNTs at higher pressure was performed at the same conditions described above except that after 45 min of the process, the gas pressure was increased up to 2 bars and the process continued additionally for 20 min.

Various techniques were employed in order to characterize the prepared CNTs. Raman spectroscopy was performed using the Renishaw spectrometer with a laser wavelength of 514 nm (Renishaw, Wotton-under-Edge, UK). A JEOL 2010 transmission electron microscope (JEOL Ltd., Akishima, Tokyo, Japan) operated at 200 kV was used to study the microstructure. A DRON-3M X-ray diffractometer with CuK source was employed to collect X-ray diffraction [XRD] spectra (Bourevestnik Inc., St. Petersburg, Russia). The Mössbauer spectra were collected in transmission geometry at room temperature using the MS2000 spectrometer with 57Fe/Rh source (40 mCu) (Belarusian State University, Minsk, Belarus). Magnetization of synthesized CNTs was measured by a ponderomotive method [20]. Calorimetric measurements were performed with a Mettler-Toledo instrument TGA/DSC-1/1600 HF (Mettler-Toledo, Inc., Greifensee, Switzerland) setup in dry air flow and at a heating rate of 1°C/min.

## Results

**Figure 2:**
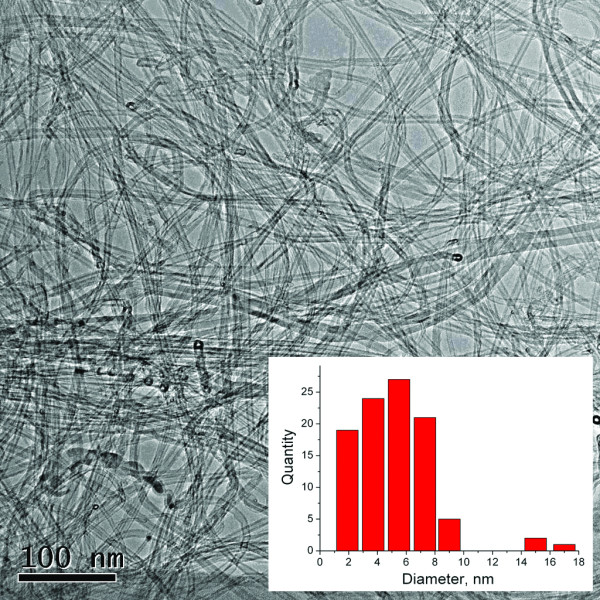
**TEM image of as-prepared CNTs**. The inset shows the histogram of the diameter distribution of CNTs.

**Figure 3:**
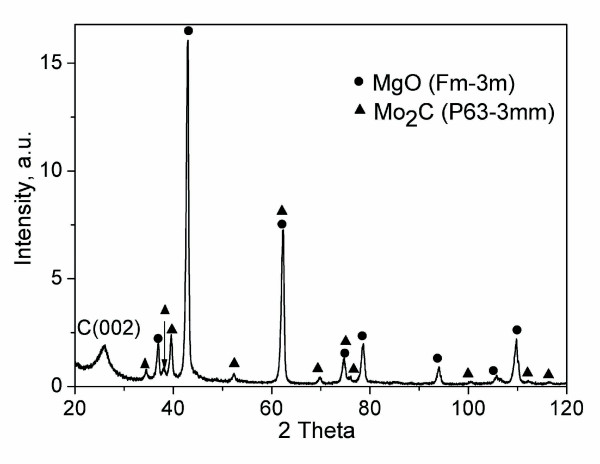
**XRD spectrum of CNTs**.

The as-synthesized product was studied by transmission electron microscopy [TEM] (Figure 2). The image shows mostly the impurity-free CNTs. Only a small amount of catalyst particles introduced to the inner space of nanotubes is visible. The diameters of CNTs are mostly in the range of 2 to 10 nm (Figure 2, inset). However, larger CNTs (approximately 20 nm) can also be observed. The phase analysis of a non-purified CNT was performed by an X-ray diffraction technique. The XRD spectrum measured in a wide range of angles is plotted in Figure 3. The broad diffraction peak at Bragg angle 2*θ *of approximately 26° corresponds to the (002) peak of the hexagonal graphite structure. The other peaks are attributed only to the MgO (Fm/3 m) and Mo_2_C (P63/3 mm) phases; no other phases were noticed. The absence of any other peaks assigned to iron or iron-containing phases could be explained by the small amount of these phases which cannot be detected owing to the resolution limit of the used XRD setup.

**Figure 4:**
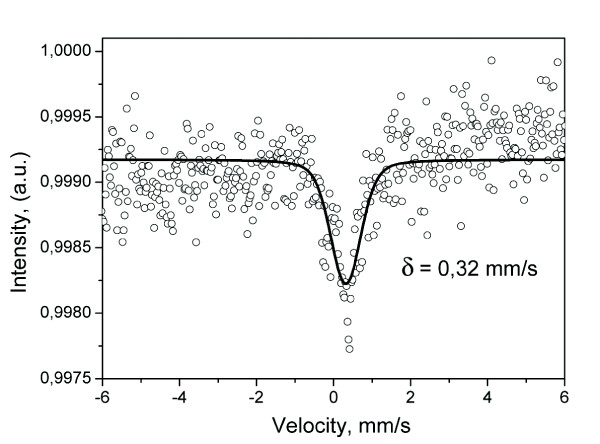
**The Mössbauer spectrum of fabricated CNTs**.

However, the Mössbauer spectroscopy allows the study of the local iron ion structures. The hyperfine interaction parameter, isomer shift [IS], deduced through the Mössbauer (Figure 4) spectrum is *δ *= 0.32 mm/s. From the value of the IS parameter, one might conclude the presence of Fe-C chemical bonds, where iron ions are in the state of Fe^3+^. We would like to emphasize that the local surroundings of iron ions have high symmetry as what appears from the fact of a single peak in the Mössbauer spectrum.

**Figure 5:**
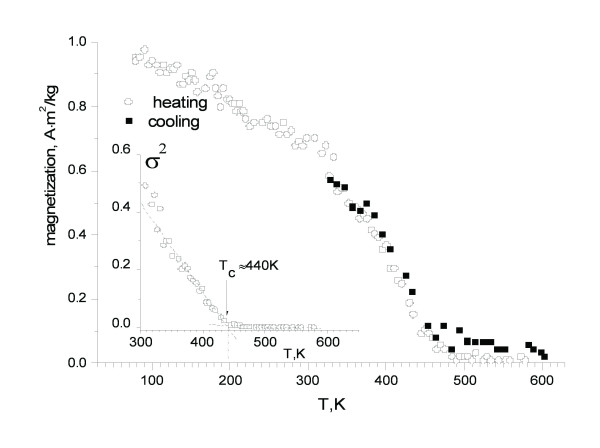
**The magnetization measurements of CNTs**. The inset shows *M*^2 ^vs. *T *plot for Curie temperature evaluation.

The magnetization measurements show the presence of magnetic phases. The *M*(*T*) curves measured in the zero field in the heating and cooling modes are presented in Figure 5. The magnetic phase has the Curie temperature [*T*_C_] of approximately 440 K. Taking into account both the Mössbauer measurements and *M*(*T*) dependence, the magnetic phase was assigned to cementite, Fe_3_C. The decreased *T*_C_, as compared to the bulk value (approximately 480 K), could be explained by the small size of Fe_3_C particles.

**Figure 6:**
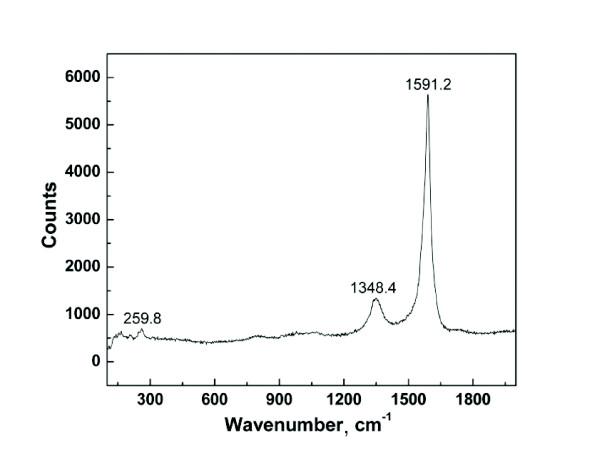
**Raman spectrum of CNTs**.

The Raman spectrum of as-grown CNTs is shown in Figure 6. The G line, which is attributed to the twice-degenerated deformation oscillations of the hexagonal ring in the *E*_2 g _electronic configuration of *D*^4^_6 h _crystal symmetry, and the D line, corresponding to the ruinous hexagonal lattice and not fully ordered forms of carbon structure, are located at 1,591 cm^-1 ^and 1,348 cm^-1^, respectively. The integrated area ratio *A*_D_/*A*_G _between the D and G bands indicates good crystalline quality of the as-grown nanotubes.

There are few peaks in the range below 300 cm^-1 ^in the Raman spectrum. Commonly, peaks in this range are assigned to the radial breathing mode [RBM] of SWCNTs. Using the simple inverse relation *v *= 9 +235/*d *(where *ν *is the frequency in units of inverse centimeter, *d *is diameter of nanotubes in nanometers), we estimated the diameters of the nanotubes in the range of 0.9 to 1.7 nm; the larger diameters cannot be estimated from the spectrum because of the spectrometer frequency cutoff. Besides, these peaks in the range below 300 cm^-1^, apart from the RBM mode of SWNTs, can be attributed to the RBM mode of the inner tubes of FWNTs [21].

**Figure 7:**
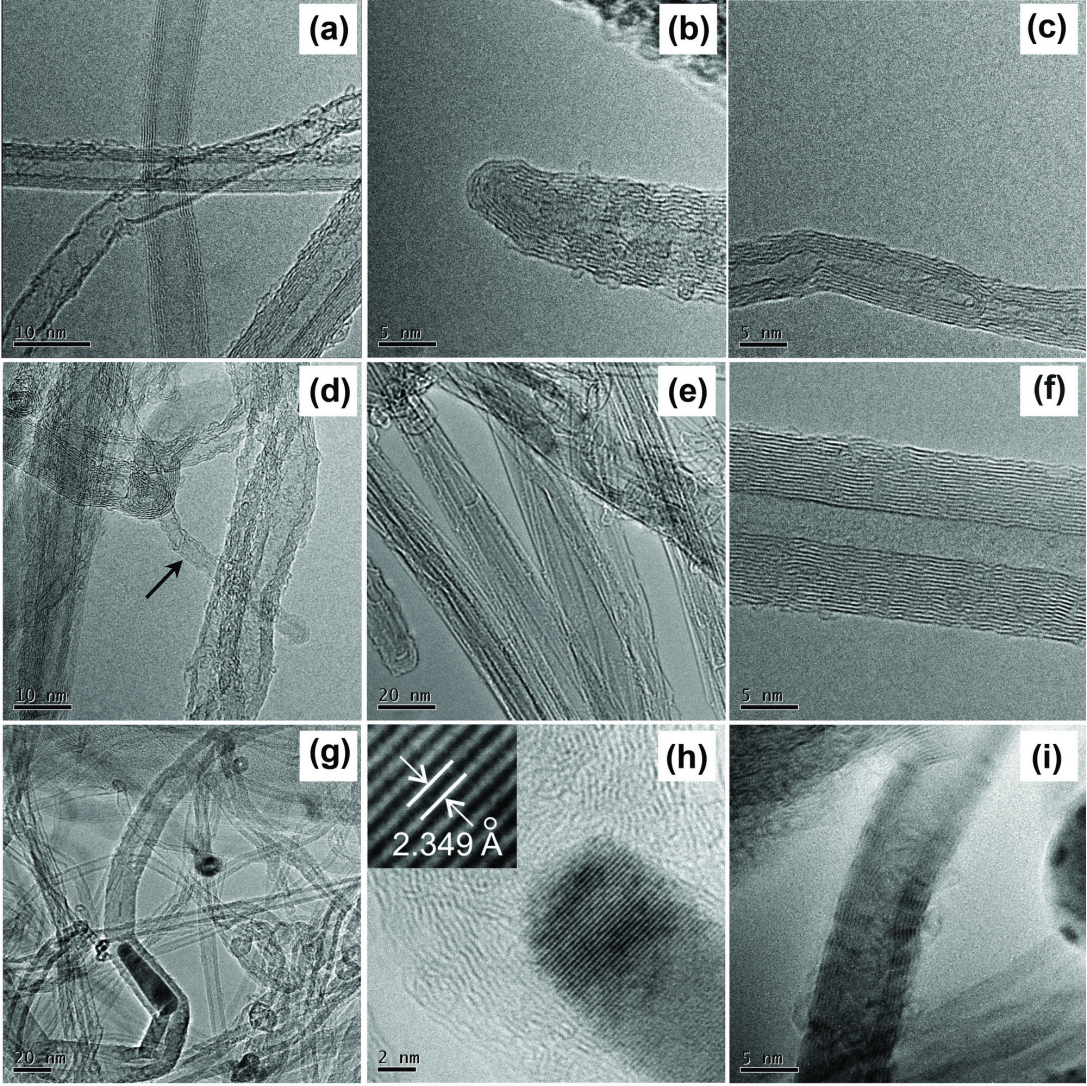
**HRTEM images of selected CNTs**. (**a**) Few-wall and multi-wall CNTs of various diameters, (**b**) a closed-tipped CNT, (**c**) CNT having a bamboo-like structure, (**d**) a double-wall CNT (indicated by an arrow), (**e**) CNT bundles, (**f**) a single CNT demonstrating a good crystallinity of the walls' structure, and (**g**, **h**, **i**) CNTs with the catalyst inclusions. The inset in Figure 7h shows a zoomed part of the encapsulated particle with the marked interplane distance.

Figure 7 represents the high-resolution TEM [HRTEM] images of CNTs. As it is seen from Figure 7a, both the few-wall and multi-wall carbon nanotubes of different diameters can be found. They usually have 3 to 10 walls and are closed-tipped (Figure 7b). A bamboo-like CNT structure is presented in Figure 7c. As shown in Figure 7d, double-wall nanotubes of relatively large diameters are also found in the array. As shown in Figure 7e, besides the single CNTs, CNT bundles can also be found. Figure 7g, h, i shows the presence of metal inclusions in CNTs. As shown in Figure 7g, a large aspect ratio of metal inclusions can be found in the nanotube channels. As shown in Figure 7h, inclusions have a single crystal structure. The accurate measurement of the crystal interplane distance of the inclusion performed using Fourier transform gives a value of approximately 0.2349 nm (see the inset), which is very close to the Fe_2_MoC (241) interplane distance (0.2345 nm). Finally, beside the central channels, metallic inclusions can also be found inside the CNT walls as well (Figure 7i).

**Figure 8:**
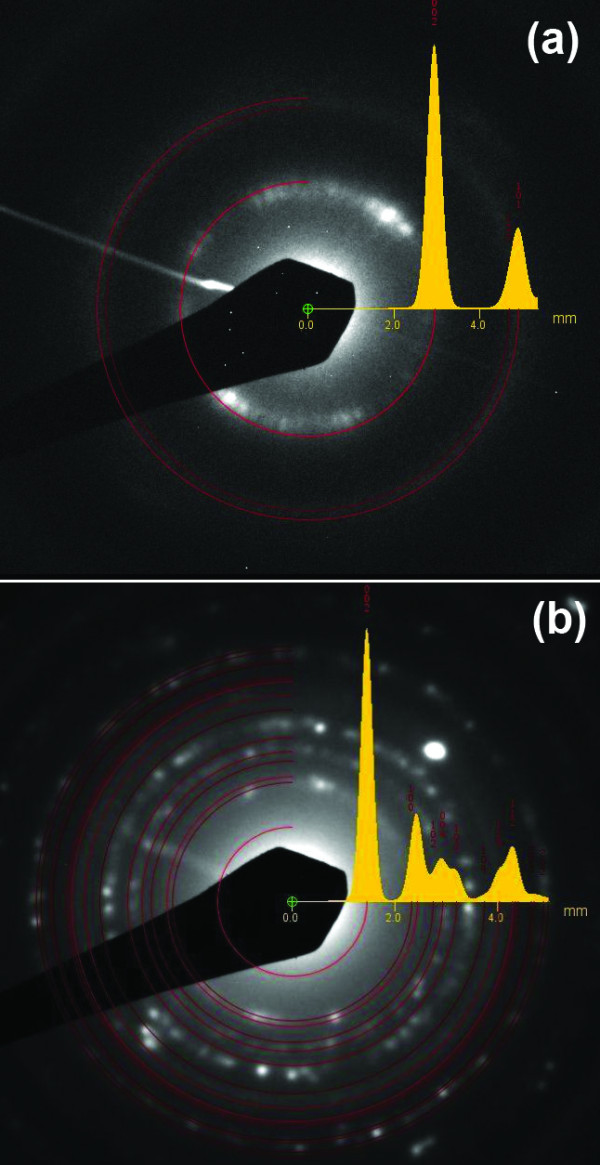
**Selected area electron diffraction images of CNTs**. (**a**) Impurity-free CNTs and (**b**) CNTs with encapsulated nanoparticles.

The selected area electron diffraction patterns both from the impurity-free and the area containing inclusion particles are presented in Figure 8a, b, respectively. The diffusion rings in Figure 8a are assigned to carbon nanotubes. Figure 8b shows an overlap of reflections from the carbon nanotubes and crystal structure related to the particles encapsulated into the nanotubes. The detailed analysis of the diffraction pattern allows the assumption of the presence of Mo_2_C and γ-Fe phases.

**Figure 9:**
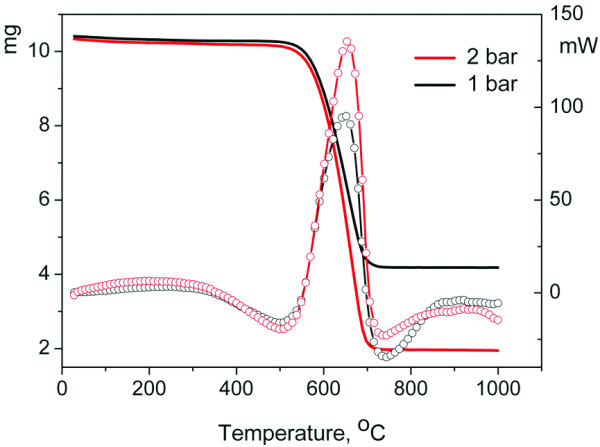
**TGA and DSC data for CNTs synthesized at different gas pressures**. The black curves corresponded to 1 bar gas pressure synthesis; the red curves correspond to 2 bar gas pressure synthesis.

The purity of the product was investigated by thermogravimetric analysis [TGA]. TGA thermogram curves of the as-prepared CNTs (Figure 9, left axis, black) demonstrates that carbon content is approximately 64% of the total mass of the product. The thermogram curve (Figure 9, left axis, red) for CNTs synthesized at the same conditions, except that after 45 min, the gas pressure was increased up to 2 bars for another 20 min, showed the increasing carbon content of up to 83%. Differential scanning calorimetry curves (Figure 9, right axis) for both pressures showed only one exothermic transition peak at approximately 650°C. It is known that amorphous carbon burns at temperatures lower than 580°C to 600°C; defect-free SWNTs, at 600°C to 620°C; and pure MWNTs with 10 layers and more, at 750°C to 790°C [22]. No peaks related to amorphous phase burning were observed. The thermal analysis data are in good agreement with our Raman and TEM findings.

## Discussion

As we pointed in the introduction part, the simple and cheap technology for producing CNTs with desired geometry of carbon nanotubes opens the way to a wide range of applications of CNTs. We would like to emphasize that our technological approach does not contain any additional calcinations or reduction step after the catalyst preparation. Besides, catalyst preparation process is consuming a lot less time; the elimination of a calcination step prevents the large-size catalytic particle formation, thus limiting the diameter distribution of CNTs in the final product. Although iron nanoparticles are initially in the oxide state, the presence of methane partially reduces the oxides, which leads to the enhancement of the methane decomposition by the presence of metal particles. Bimetallic catalysts show better methane decomposition performance, and the decomposition rate depends on the Fe/Mo ratio [23]. In our work the Fe/Mo ratio is optimized for few-wall nanotube synthesis.

Moreover, it was revealed that reaction with methane not only reduces the iron oxide to iron, but also turns some fraction of it into cementite [24], which is reported to be not catalytically active for methane/acetylene decomposition [23]. From the magnetic measurement data and Mössbauer spectrum, we conclude that most of the nanoparticles are in Fe_3_C phase in the final product. Only the selected area electron diffraction data indicated the presence of some amount of γ-Fe phase. We do believe that some part of cementite observed by our *ex-situ *measurements was formed during the cooling process when carbon interacting with the γ-Fe particles transited to the cementite phase [25].

Growth of CNTs is considered as a multistep process involving formation of Fe_3_C from iron catalyst nanoparticles [26-28]. Formation of CNTs from cementite was demonstrated by *in-situ *TEM measurements [29]. However, there are still debates whether Fe_3_C could promote formation and growth of very thin CNTs [15]. In general, catalytic activity and carbon solubility of catalytic species depend on the temperature, diffusion, and particle size. Thus, catalyst species could be active or inactive depending on the carbon atom kinetics. Molybdenum, due to the higher carbon solubility as compared to iron [30], can serve as a regulator of carbon atom kinetics for neighboring γ-Fe and Fe_3_C catalyst species, preventing their poisoning at the conditions we perform our synthesis. The Fe_2_MoC phase observed using HRTEM in our experiment claimed to be inactive for CNT growth [15]. That phase most probably arises from the Fe_x_Mo_y_O_z _phase, and its formation is limited due to the absence of calcination. The low amount of Fe_2_MoC can be explained this way (Mössbauer and XRD spectra do not show any evidence of this phase).

Let us briefly discuss the impact of synthesis pressure on CNT yield. It was revealed that prolongation of reaction time in our experiments for more than 45 min did not increase significantly the mass of the CNT product. That fact usually contributed to the diffusion issue of carbon feedstock molecules through the mat of CNTs [31]. However, it was found that increasing the pressure up to 2 bars in the furnace during CNT synthesis, as was described in the experimental part, leads to the CNT-yield increase approximately by 20 wt.% (see Figure 9, red curve). Thus, we attribute the longer catalytic activity and, as a consequence, the higher CNT yield at the realized synthesis conditions to the increasing hydrocarbon molecular diffusivity as the gas pressure increases.

## Conclusions

The technological approach for the simple high-yield CVD growth of few-wall carbon nanotubes was developed. The Fe-Mo-MgO catalyst with 1:8:40 molar ratio was prepared by impregnation method from purum quality components without any calcinations and reduction steps. TEM, Raman, and thermogravimetrical studies showed that decomposition of methane/acetylene at 900°C over the catalyst leads to the formation of CNTs with diameters in the range of 2 to 10 nm. The phase of catalytic species in CNT was investigated by *ex-situ *Mössbauer, XRD, HRTEM, and electron diffraction techniques. The phase transitions of metallic components of binary catalyst and their role in the CNT growth process were discussed. It was demonstrated that slightly increasing the gas pressure during the CNT growth leads to the increase in the CNT yield.

## Abbreviations

CNT: carbon nanotube; CVD: chemical vapor deposition; DSC: differential scanning calorimetry; FEC: field-emission cathode; FWNT: few-wall carbon nanotube; HRTEM: high-resolution transmission electron microscopy; MWNT: multi-wall carbon nanotube; SWNT: single-wall carbon nanotube; TEM: transmission electron microscopy; TGA: thermogravimetric analysis; XRD: X-ray diffraction.

## Competing interests

The authors declare that they have no competing interests.

## Authors' contributions

VAL coordinated the study, analyzed the data, and wrote the manuscript. BGS carried out the calorimetric measurements and analyzed the data. ASB and YPS performed the synthesis of CNTs. IK and AP analyzed the data and wrote the article. BKT and MS carried out the TEM and Raman measurements. All authors read and approved the final manuscript.
